# The role of IL-6 in coronavirus, especially in COVID-19

**DOI:** 10.3389/fphar.2022.1033674

**Published:** 2022-11-23

**Authors:** Xinyi Wang, Guozheng Tang, Yuchen Liu, Lizhi Zhang, Bangjie Chen, Yanxun Han, Ziyue Fu, Liuning Wang, Guangzhi Hu, Qing Ma, Shuyan Sheng, Jianpeng Wang, Xinyang Hu, Song Shao

**Affiliations:** ^1^ Department of Radiation Oncology, The First Affiliated Hospital of Anhui Medical University, Hefei, Anhui, China; ^2^ Department of Orthopaedics, Lu’an Hospital of Anhui Medical University, Lu’an, Anhui, China; ^3^ Department of Otolaryngology, Head and Neck Surgery, The First Affifiliated Hospital of Anhui Medical University, Hefei, Anhui, China; ^4^ First Clinical Medical College, Anhui Medical University, Hefei, Anhui, China; ^5^ Department of Oncology, The First Affiliated Hospital of Anhui Medical University, Hefei, Anhui, China; ^6^ Second Clinical Medical College, Anhui Medical University, Hefei, Anhui, China

**Keywords:** IL-6, COVID-19, SARS, MERS, coronavirus

## Abstract

Severe Acute Respiratory Syndrome Coronavirus 2 (SARS-CoV-2) infects both people and animals and may cause significant respiratory problems, including lung illness: Corona Virus Disease 2019 (COVID-19). Swabs taken from the throat and nose of people who have the illness or are suspected of having it have shown this pathogenic virus. When SARS-CoV-2 infects the upper and lower respiratory tracts, it may induce moderate to severe respiratory symptoms, as well as the release of pro-inflammatory cytokines including interleukin 6 (IL-6). COVID-19-induced reduction of IL-6 in an inflammatory state may have a hitherto undiscovered therapeutic impact. Many inflammatory disorders, including viral infections, has been found to be regulated by IL-6. In individuals with COVID-19, one of the primary inflammatory agents that causes inflammatory storm is IL-6. It promotes the inflammatory response of virus infection, including the virus infection caused by SARS-CoV-2, and provides a new diagnostic and therapeutic strategy. In this review article, we highlighted the functions of IL-6 in the coronavirus, especially in COVID-19, showing that IL-6 activation plays an important function in the progression of coronavirus and is a rational therapeutic goal for inflammation aimed at coronavirus.

## Introduction

Severe Acute Respiratory Syndrome Coronavirus 2 (SARS-CoV-2), a betacoronavirus closely related to Middle East Respiratory Syndrome Coronavirus (MERS-CoV) and Severe Acute Respiratory Syndrome Coronavirus (SARS-CoV), the organisms responsible for Middle East respiratory sickness (MERS) and severe acute respiratory syndrome (SARS), respectively (COVID-19). MERS-CoV and SARS-CoV cause high mortality, with the majority of cases resulting from an inflammatory viral pneumonia that progresses to acute respiratory distress syndrome 1 (ARDS1). ARDS2 was detected in 81 percent of fatal patients infected with COVID-19. In light of this, a recent letter published in The Lancet recommends that all COVID-19 patients be examined for hyperinflammation to identify those who may benefit from immunosuppression or immunomodulation to prevent acute lung injury (ALI). The coronavirus family consists of four “established” human coronaviruses (HCOVs), two of which have been identified since the 1960s: HCOV-OC43 and HCOV-229E. These two viruses produce a milder respiratory illness and, after rhinoviruses, are the most frequent cause (10–30 percent) of the common cold (Hamre and Procknow, 1966; McIntosh et al., 1967; van der Hoek, 2007). Following increased coronavirus screening, two new HCoVs, HCoV-NL63 and HCoV-HKU1, were found lately (van der Hoek et al., 2004; Woo et al., 2005). Recent research suggests that HCoV-NL63, -229E, and -OC43 are also the result of zoonotic transmission from bats (de Wilde et al., 2018).

Considering the importance of interleukin 6 (IL-6) in airway disease, preliminary studies using humanized monoclonal antibodies against the IL-6 Receptor (Tocilizumab) to target this cytokine therapeutically in response to COVID-19 infection have shown promising results, but additional research is required. It has been shown that the antimalarial drug hydroxychloroquine (Plaquenil) inhibits the expression of toll-like receptors (TLRs) and the production of IL-6, and hence may have an anti-COVID-19 impact (Wang et al., 2020).

## Overview of IL-6

IL-6 is a prototype cytokine with pleiotropic activity and functional redundancy that is necessary for host defense (Akira et al., 1993; Tanaka and Kishimoto, 2014; Tanaka et al., 2016a). Due to infection and tissue damage, IL-6 is produced rapidly by a variety of cells, including immune-mediated cells, mesenchymal cells, endothelial cells, fibroblasts and cancer cells, and even many other cells, which promotes host defense by stimulating acute phase reactions, hematopoiesis, and immune responses (Akira et al., 1993; Tanaka et al., 2014; Tanaka et al., 2016a). Because these processes are necessary for the elimination of pathogenic microorganisms and tissue healing, IL-6 is a crucial cytokine in host defense. Monocytes and macrophages produce IL-6 in response to infections or tissue injuries by stimulating pattern recognition receptors with pathogen-associated molecular patterns (PAMPs) or damage-associated molecular patterns (DAMPs) and serum IL-6 levels rise to several tens to hundreds of pg/ml, depending on the infection or injury, but in healthy condition, it is not higher than 4 pg/ml (Tanaka et al., 2012; Tanaka et al., 2014; Kang et al., 2015).

### The role of IL-6 in inflammation

The purpose of acute inflammation is to transport white blood cells (neutrophils, lymphocytes, monocytes, etc.) and plasma proteins (complements, antibodies, etc.) to the inflammatory site to kill and remove inflammatory factors. In addition to these cells collected from local lesions, various cytokines, such as IL-6 and tumor necrosis factor-α (TNF-α), are also produced during inflammation (Kaur et al., 2020)**.** In acute inflammation, IL-6 enters the liver through blood and induces a large number of acute phase proteins, such as C-reactive protein (CRP), serum amyloid A (SAA), etc. At the same time, the abnormal synthesis of IL-6 also plays a pathological role in chronic inflammation and autoimmunity. When the high concentration of SAA persists, it will promote the generation of chronic inflammatory disease complications and organ failure (Heinrich et al., 1990; Gillmore et al., 2001; Tanaka et al., 2014)**.** Persistent acute inflammation will develop into chronic inflammation, which will eventually lead to tissue damage. IL-6 is an important regulator for the transformation of inflammation from the acute phase to the chronic phase.

### The role of IL-6 in disease

IL-6 can mediate a variety of signaling pathways, regulate cell proliferation, differentiation, apoptosis, angiogenesis and metastasis, and play a role in a variety of diseases. In rheumatoid arthritis (RA), the expression of TLRs signaling pathway can be used as the activation pathway of IL-6. IL-6 plays a key role in osteoclast mediated bone resorption. In RA patients, the levels of IL-6 and IL-6R in serum and synovial fluid of affected joints are elevated (Pandolfi et al., 2020). STAT3 pathway is considered to be an important signal transducer downstream of gp130 signal. STAT3 itself is a carcinogenic gene and plays a key role in connecting inflammation and cancer. It can participate in tumor angiogenesis by up regulating the expression of matrix metalloproteinase-9 (MMP-9) (Kujawski et al., 2008; Yu et al., 2009; Kishimoto, 2010).

### Signaling pathway of IL-6

IL-6 interacts with its specific receptor IL-6R, and its complex IL-6/IL-6R interacts with and activates gp130, which is a signal transducer shared by the IL-6 family of cytokines. The hexamer complex composed of IL-6/IL-6R/gp130 performs various physiological and biochemical functions of IL-6 by activating different signal pathways (including classic signaling and trans-signaling).

The combination of IL-6 and membrane-bound IL6R (mIL-6R) can mediate classic signaling, while the combination of IL-R and soluble IL-6R (sIL-6R) can mediate trans-signaling. The hexamer complex composed of IL-6/IL-6R/gp130 first activates Janus kinase (JAK) and starts the enzymatic reaction. Two common pathways include JAK/STAT pathway and SHP-2/ERK pathway. Src homology phosphotyrosine phosphatase 2 (SHP-2) connects to mitogen-activated protein kinase (MAPK), phosphorylates growth factor receptor-bound protein 2 (GRB2) associated binding protein 1 (Gab1), and transfers it to the cell membrane to coordinate the ongoing activation of MAPK and phosphatidylinositol 3-kinase (PI3K). PI3K/Akt pathway contributes to the activation of nuclear factor kappa-B(NF-κB). (Heinrich et al., 2003; Mihara et al., 2012; Tanaka et al., 2014; Baran et al., 2018; Jiang et al., 2021).

When the level of IL-6 in serum is low, the classic signaling pathway plays a leading role, which can play an anti-inflammatory role; When the concentration of IL-6 increases, IL-6R starts trans-signaling transduction, and proinflammatory reaction occurs in a wider cell population (Lee et al., 2014).

### Specificity of IL-6

IL-6 is an important member of the cytokine network, a central mediator of cytokine release syndrome (CRS) toxicity, and plays a central role in acute inflammatory response (Lee et al., 2014). IL-6 can induce the production of CRP and procalcitonin (PCT), which is directly related to inflammation and infection, facilitating the diagnosis of early inflammation and early warning of sepsis. Therefore, IL-6 can be used as a biomarker of disease severity and prognosis in CRS. A large number of clinical data show that CRS is related to the severity of COVID-19 and is the key cause of severe COVID-19 ^26–28^. A variety of cytokines, including IL-6, are involved in severe COVID-19, and anti-inflammatory treatment is of great significance in the protection of severe patients. Therefore, the role of IL-6 in COVID-19 is irreplaceable by other cytokines.

## The function of IL-6 in SARS-CoV and MERS-CoV

### IL-6 in SARS-CoV

From 2002 to 2003, SARS-CoV, a new coronavirus, caused a severe respiratory epidemic worldwide (Yu et al., 2019). SARS is characterized by influenza-like symptoms, a high fever, myalgia, dyspnea, lymphopenia, and severe breathing problems caused by lung infiltrates (pneumonia) (De Clercq, 2006). The N proteins and RNA of SARS-CoV were found in lung, bronchial epithelial cells and macrophages, suggesting that these cells may be infected with SARS-CoV. Monoclonal antibodies against MCP-1 and TGF-1, as well as monoclonal antibodies against IL-6, substantially interacted with the angiotensin converting enzyme 2 (ACE2) and the S proteins of SARS-CoV produced by most cells (He et al., 2006). Sheng et al. (Sheng et al., 2005) collected information on SARS-associated coronavirus-infected hospitalized patients in Taiwan University Hospital, and determined a series of plasma inflammatory cytokines, including IL-1β, IL-6, IL-8 and TNF-α. The fast rise of the inflammatory cytokines IL-6, IL-8, and TNF-α was shown to be associated with the development of SARS-associated ARDS. The significance of IL-6 in the acute phase of SARS, however, remained unknown.

The IL-6 cytokine’s mRNA expression was observed to be higher in SARS patients’ peripheral blood mononuclear cell (PBMC) (Yu et al., 2005; Dosch et al., 2009). After sufficient immunosuppressive medication, the levels of IL-6 and TNF in the acute phase grew dramatically and recovered to normal, according to a study of SARS-CoV infected patients (Hsueh et al., 2004). Wang et al. (Bai et al., 2008) found that the S proteins of SARS-CoV was involved in the synthesis of pro-inflammatory cytokine during the virus-host cell contact stage. IL-6 is one of the primary cytokines released by activated macrophages in excess amounts. The level of IL-6 expression was found to be greater in SARS patients and was associated with the severity of their sickness (Liu et al., 2020a). Before and during the treatment of many early SARS patients, the amount of IL-6 and TNF-α induced by T cells or monocyte activators was higher than the normal value, and some people still increased after treatment. This indicated that SARS may cause long-term imbalance of cytokines. Future research should focus on improving antiviral therapy and trying to use relevant cytokine inhibitors to limit damage (Jones et al., 2004).

### IL-6 in MERS-CoV

In 2012, MERS-CoV was discovered and causes a spectrum of severe respiratory disease known as MERS. There were 1,728 confirmed MERS infections in 27 countries as of 26 April 2016, with 624 deaths (Wise, 2012; Zaki et al., 2012; Hijawi et al., 2013; Korea Centers for Disease, 2015; de Wit et al., 2016) (http://www.who.int/csr/don/26-april-2016-mers-saudi-arabia/en/ (2016)).

MERS is most commonly referred to as lower respiratory tract (LRT) disease and involves cough, fever, dyspnea, and pneumonia. Between 20 and 40 percent of infected individuals may develop ARDS, multiple organ failure and death. MERS is another fatal zoonotic coronavirus illness, comparable to SARS; it causes respiratory failure and severe kidney damage (also has the effect on the growth of kidney cells under laboratory conditions). Patients with underlying diseases have been reported more frequently and more fatal. The majority of human MERS infections are linked to medical institution infection prevention and control (IPC) failures. According to reports, the detection rate of all viruses detected in health care workers (HCWs) is about 20%.

Rossigno et al. (Rossignol, 2016) reported that oral administration of 100 mg/kg nitazoxanide 2 h prior to a 1 ml intraperitoneal injection of 4 percent thioglycollate (TG) decreased plasma IL-6 levels by 90 percent compared to vehicle-treated mice 6 h after TG administration. Although the clinical relevance of these results has not been determined, they suggest that nitazoxanide may improve the prognosis of MERS-CoV patients by reducing the overproduction of pro-inflammatory cytokines such as IL-6. According to recent research, the S protein of MERS-CoV does not increase the production of TNF or IL-6, but rather suppresses their generation by Lipopolysaccharide (LPS). This shows that the activation of these factors found in previous research was related to active viral replication, since macrophages were infected with an active virus at the time (Al-Qahtani et al., 2017). IL-6 expression was elevated in severe MERS-CoV infections compared to moderate ones (Liu et al., 2020a). Nitazoxanide is a broad-spectrum antiviral drug with *in vitro* activity against coronavirus, which can be used to treat viral respiratory infection and inhibit the production of IL-6 (Rossignol, 2016). A phase 2b/3 clinical trial by Haffizulla et al. (Haffizulla et al., 2014) showed that nitazoxanide 600 mg twice a day for five consecutive days was related to the shortening of the duration of symptoms in patients with acute non complex influenza. At present, nitazoxanide is a potential drug to treat MERS, so it is of great significance to evaluate its therapeutic effect when used alone or in combination with other candidate drugs such as oseltamivir (Rossignol et al., 2009).

## IL-6 in COVID-19

In December 2019, COVID-19 first came into public view. Globally, as of 6: 49p.m. Central European summer Time (CEST), 26 August 2022, there have been 596, 873, 121 confirmed cases of COVID-19, including 6, 459,684 deaths, reported to World Health Organization (WHO). As of 23 August 2022, a total of 12, 449,443,718 vaccine doses have been administered (https://covid19.who.int/). The nucleic acid sequences of COVID-19 are coronavirus-specific, and they vary from the known human coronavirus specializations. These sequences are identical to those found in severe SARS or MERS coronavirus. The combination of the S protein and ACE2 in COVID-19 provides a severe public health danger to human transmission (Xu et al., 2020; Hu et al., 2021b).

**FIGURE 1:**
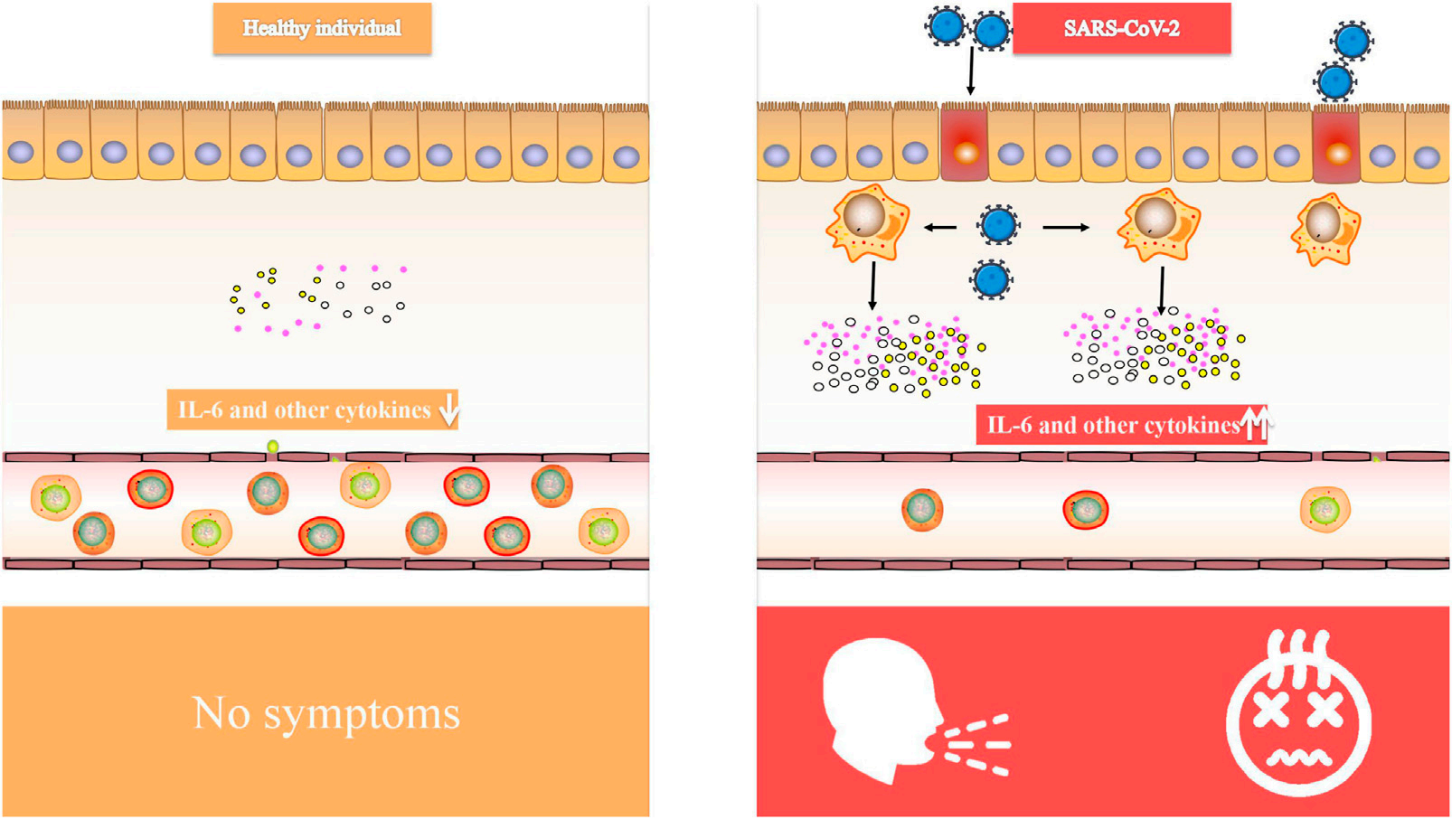
Comparison of IL-6 and other cytokines in healthy individual and SARS-CoV-2 infected people.

Critical patients with COVID-19 had increased plasma levels of cytokines, similar to SARS, suggesting that an inflammatory storm is involved in illness development (Luo et al., 2020). Inflammatory cytokine (IL-6, IL-1, and IFN) blockade, stem cell therapy, immune cell reduction, transfusion of convalescent plasma, and artificial extracorporeal liver support are all potential therapies for COVID-19 (Al-Qahtani et al., 2017), and we believe that IL-6 blockade is a viable technique for COVID-induced CRS. CRS is a systemic inflammatory response defined by a rapid rise in a high number of pro-inflammatory cytokines (Teijaro, 2017; Norelli et al., 2018; Shimabukuro-Vornhagen et al., 2018), which may be induced by infection, certain medications and other situations. CRS is more frequent in immune-related conditions and treatments, such as chimeric antigen receptor-T (CAR-T) therapy, organ transplantation sepsis (Chousterman et al., 2017) and viral infections. We observed that elevated IL-6 levels were consistently reported in several COVID-19 studies (Huang et al., 2020; Lai et al., 2020; Xu et al., 2020; Hu et al., 2021b), suggesting that it might be used as a disease severity predictor (Luo et al., 2020). In patients with COVID-19, IL-6 levels were linked to death in a large retrospective cohort study (Tanaka et al., 2012). In the dendritic cell-T cell interaction, IL-6 is required for the production of T helper 17 (Th17) cells (Mackay and Arden, 2015). According to Xu et al. (McIntosh et al., 1967) elevated IL-6 might account for the highly active Th17 cells seen in COVID-19 patients. Animal investigations of SARS-CoV have shown that suppressing NF-κB, a critical transcription factor of IL-6, or infecting animals with SARS-CoV missing the coronavirus envelope (E) protein, a potent stimulant to NF-κB signaling, enhanced animal survival with lower IL-6 levels (Zhang et al., 2020). The E proteins of SARS-CoV-2 (Ref sequence QHD43418.1) and SARS-CoV (Ref sequence NP 828854.1) are 95 percent similar, as found. Given that the E protein is a virulence determinant and mediates the coronavirus immune response (Kai and Kai, 2020; Moore and June, 2020), it is reasonable to presume that both viruses provoke an identical immune response. Therefore, targeting IL-6 for COVID-induced CRS may be advantageous (Figure 1).

Patients with severe COVID-19 had a greater IL-6/IFN ratio than those with mild COVID-19, which might be due to a stronger cytokine storm promoting lung injury (Lagunas-Rangel and Chavez-Valencia, 2020). This raises the issue of whether IL-6 inhibition is exclusively helpful in individuals who have high IL-6 serum expression levels. If this is the case, IL-6 testing may become a necessary component of the rating system. Moreover, the expression level of IL-6 may not be sufficient to indicate its functional downstream effects. A test that distinguishes functional IL-6 from total IL-6 might be beneficial for directing treatment decisions. CRP, an acute-phase inflammatory protein generated by IL-6-dependent hepatic biosynthesis, is a reliable indicator of IL-6 bioactivity and is used to predict the severity of CRS and evaluate the success of IL-6 blocking in CAR-T cell-induced CRS patients (van der Hoek et al., 2004; de Wilde et al., 2018). Unknown is the CRP level in virus-induced CRS. With a few exceptions (Hu et al., 2021b), the majority of studies (Liu et al., 2020b; Luo et al., 2020; Smilowitz et al., 2021) found an association between elevated CRP levels and severe COVID-19. In the future, however, further biomarker research will be necessary for risk stratification and therapeutic effect monitoring. In the inflammatory network, there are several pharmacological agents that target IL-1, IL-18, TNF, and IFN, as well as JAK/STAT signaling (Table 1). Regular testing for inflammatory cytokines should be performed if these medications are effective (Liu et al., 2020a). Relevant clinical trials showed that in the study on the treatment of COVID-19 inpatients with anti-IL-6 receptor antibody tocillizumab, there was no support for the conjecture that “the use of anti-IL-6 drug intervention can improve the symptoms of COVID-19, such as hypoxia and respiratory failure, and reduce the risk of death” (Stone et al., 2020; Declercq et al., 2021). However, tocillizumab may still be effective in severe patients, so further research should be conducted in the future (Soin et al., 2021).

**TABLE 1:** Study on IL-6 in SARS-CoV-2, SARS-CoV and MERS-CoV.

Abbreviations	Terms
ACE2	angiotensin converting enzyme 2
ALI	acute lung injury
AP-1	activator protein 1
ARDS1	acute respiratory distress syndrome 1
CAR-T	chimeric antigen receptor-T
CEST	Central European summer Time
COVID-19	Corona Virus Disease 2019
CRP	C-reactive protein
CRS	cytokine release syndrome
DAMPs	Damage-associated molecular patterns
Gab1	GRB2 associated binding protein 1
GRB2	growth factor receptor-bound protein 2
GvHD	graft-versus-host disease
HCOVs	human coronaviruses
HCWs	health care workers
HMGB1	high mobility group box 1
IL	interleukin
IPC	infection prevention and control
IRF-1	interferon regulatory factor 1
JAK	Janus kinase
LPS	Lipopolysaccharide
LRT	lower respiratory tract
MAPK	mitogen-activated protein kinase
MAS	macrophage activation syndrome
MERS	Middle East respiratory sickness
MERS-CoV	Middle East Respiratory Syndrome Coronavirus
mIL-6R	membrane-bound IL6R
MMP-9	matrix metalloproteinase-9
NF-IL-6	nuclear factor IL-6
NF-κB	nuclear factor kappa-B
PAMPs	pathogen-associated molecular patterns
PBMC	peripheral blood mononuclear cell
PCT	procalcitonin
PI3K	phosphatidylinositol 3-kinase
PRRs	Pathogen-recognition receptors
RA	rheumatoid arthritis
RAGE	receptor for advanced glycation end products
SAA	serum amyloid A
SARS	severe acute respiratory syndrome
SARS-CoV	Severe Acute Respiratory Syndrome Coronavirus
SARS-CoV-2	Severe Acute Respiratory Syndrome Coronavirus 2
SHP-2	Src homology phosphotyrosine phosphatase 2
sIL-6R	soluble IL-6R
SP1	specificity protein 1
TG	thioglycollate
Th17	T helper 17
TLRs	toll-like receptors
TMPRSS2	transmembrane protease serine 2
TNF-α	tumor necrosis factor-α
WHO	World Health Organization

## Regulatory mechanisms of IL-6

**FIGURE 2:**
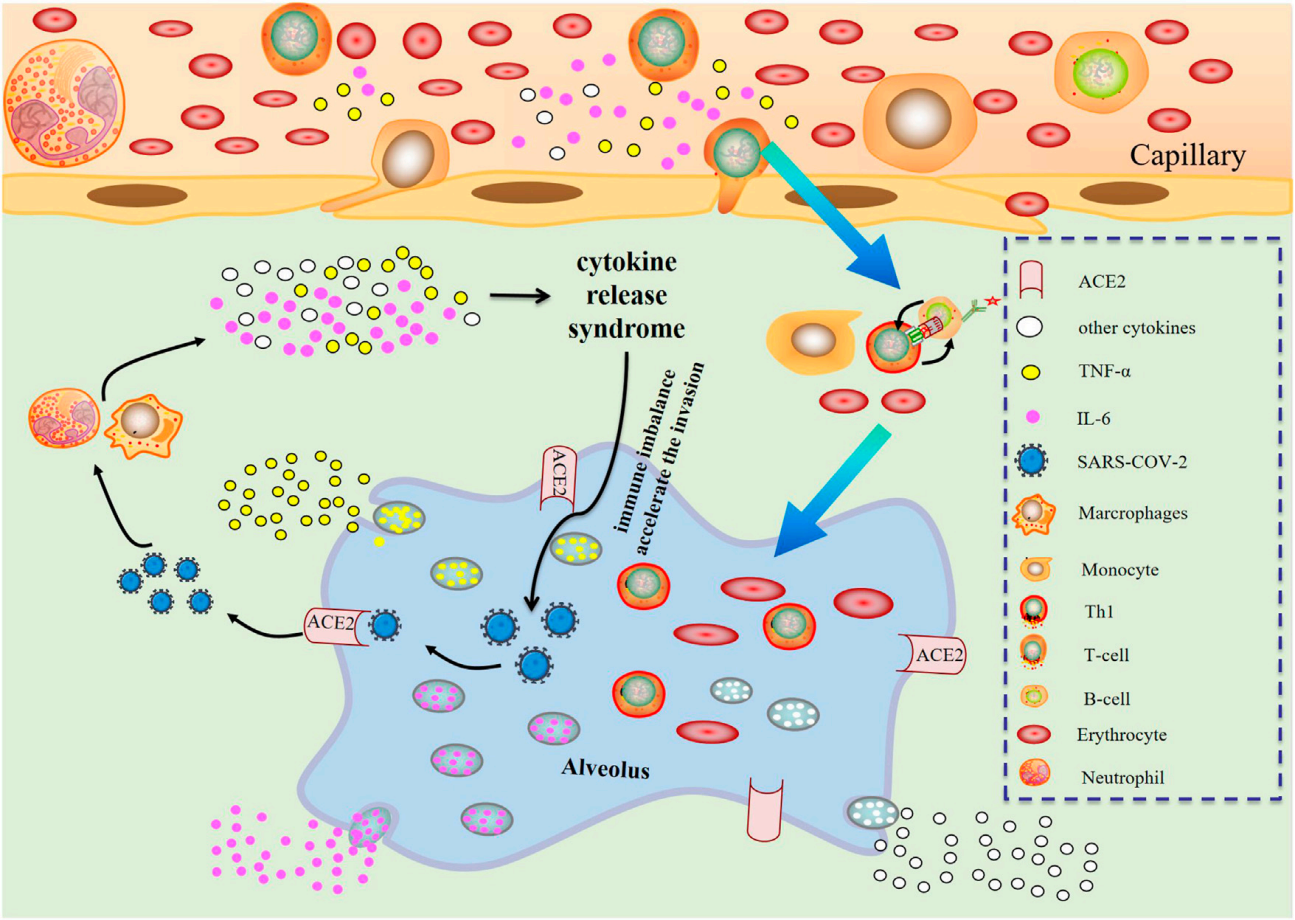
Changes of various immune cells and related substances in alveolus after SARS-CoV-2 invasion.

The possible mechanism of CRS in severe COVID-19 patients is that SARS-CoV-2 infects with alveolar epithelial cells through ACE2 receptor. The loss of epithelial cells and increased cell permeability lead to the release of the virus. SARS-CoV-2 stimulates the innate immune system, leading macrophages and other innate immune cells, including IL-6, to generate a large number of cytokines and chemokines. Antigen-presenting cells may also initiate adaptive immunity (mainly dendritic cells). T cells and B cells are antiviral cells that indirectly or directly stimulate the generation of proinflammatory cytokines. In addition, when inflammatory chemicals stimulate the alveoli, a large amount of inflammatory exudate and erythrocytes enter the alveoli, resulting in dyspnea and respiratory arrest (Figure 2).

In an infected lesion, IL-6 generates warning signals in the whole body. Pathogen-recognition receptors (PRRs) on immune cells, including monocytes and macrophages, identify PAMPs in lesions (Kumar et al., 2011). Among the PRRs are TLRs, retinoic acid-inducible gene-1-like receptors, nucleotide-binding oligomerization domain-like receptors, and DNA receptors. They stimulate the synthesis of inflammatory cytokine mRNA such as IL-6, TNF and IL-1 by activating many signaling pathways, including NF-κB. Additionally, TNF and IL-1 activate transcription factors, leading to the generation of IL-6.

In the event of tissue damage, IL-6 also transmits a warning signal. DAMPs, which are produced by dead or damaged cells in noninfectious inflammations such as burns or trauma, either directly or indirectly exacerbate inflammation. During sterile surgical operations, an increase in serum IL-6 levels precedes an increase in body temperature and serum acute phase protein concentration (Nishimoto et al., 1989). DAMPs from damaged cells include, among others, mitochondrial DNA, high mobility group box 1 (HMGB1), and S100 proteins (Bianchi, 2007). HMGB1 binding to TLR2, TLR4, and the receptor for advanced glycation end products (RAGE) may trigger inflammation; nevertheless, blood mtDNA levels in trauma patients are hundreds of times higher than in controls, causing TLR9 stimulation and NF-κB activation (Zhang et al., 2010). The S100 family consists of more than 25 proteins, some of which interact with RAGE to generate sterile inflammation (Sims et al., 2010).

**FIGURE 3:**
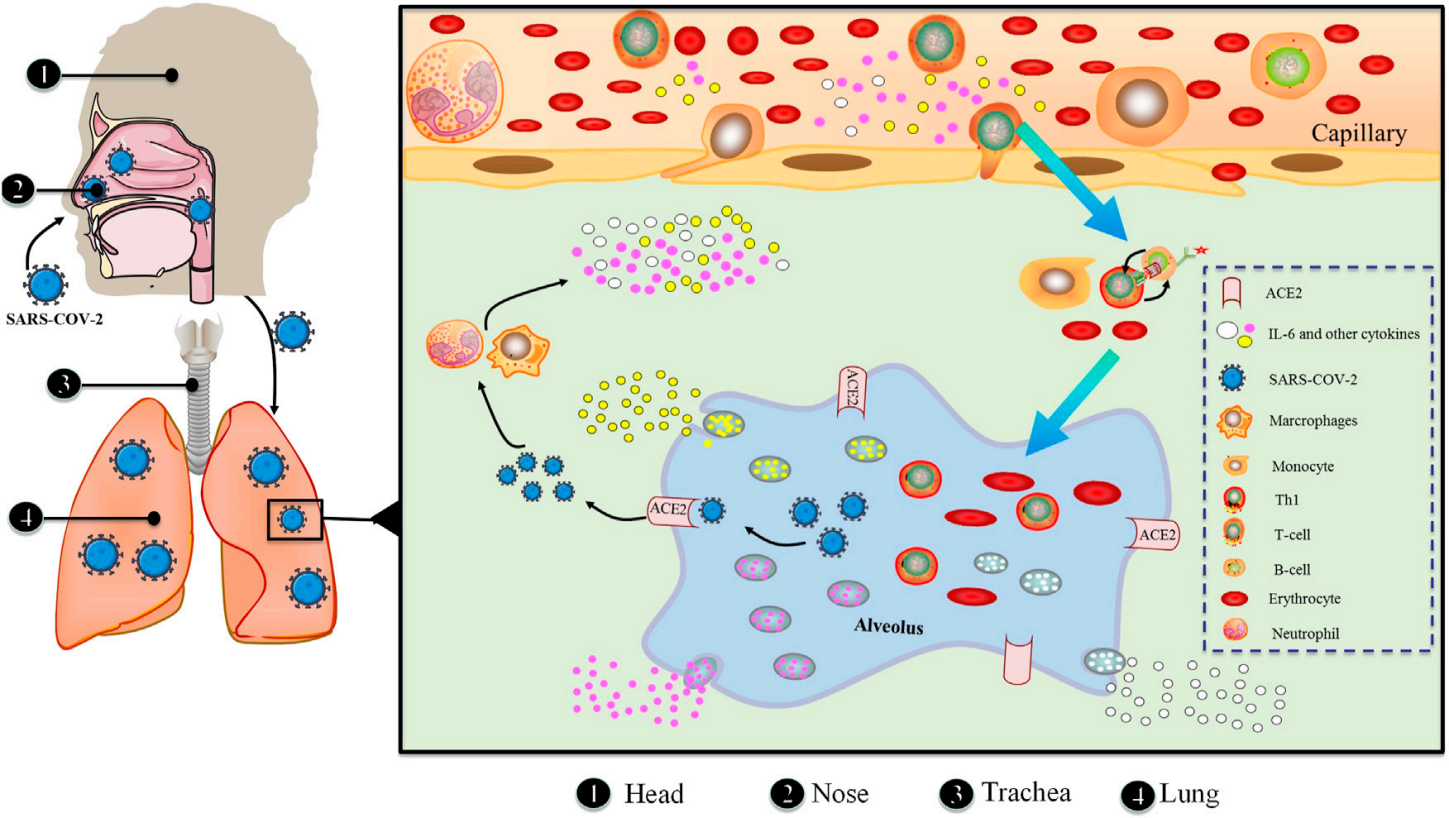
Macro and micro aspects of SARS-CoV-2 invading respiratory system.

In response to various stimuli, in addition to immune-mediated cells, IL-6 is generated by mesenchymal cells, endothelial cells, fibroblasts and a range of other cells (Akira et al., 1993). The stringent gene transcriptional and post-transcriptional regulation of IL-6 production is necessitated by the fact that IL-6 acts as a signal to alert the presence of an emergency. A multitude of transcription factors control the IL-6 gene’s transcription (Figure 3). The 5′ flanking region of the human IL-6 gene has binding sites for NF-κB, specificity protein 1 (SP1), nuclear factor IL-6 (NF-IL-6), activator protein 1 (AP-1) and interferon regulatory factor 1 (IRF-1) (Libermann and Baltimore, 1990; Akira and Kishimoto, 1992; Matsusaka et al., 1993). The IL-6 promoter is active when cis-regulatory elements are stimulated by IL-1, TNF, TLR-mediated signal and forskolin (Tanaka et al., 2014).

## The mechanism of COVID-19

**FIGURE 4:**
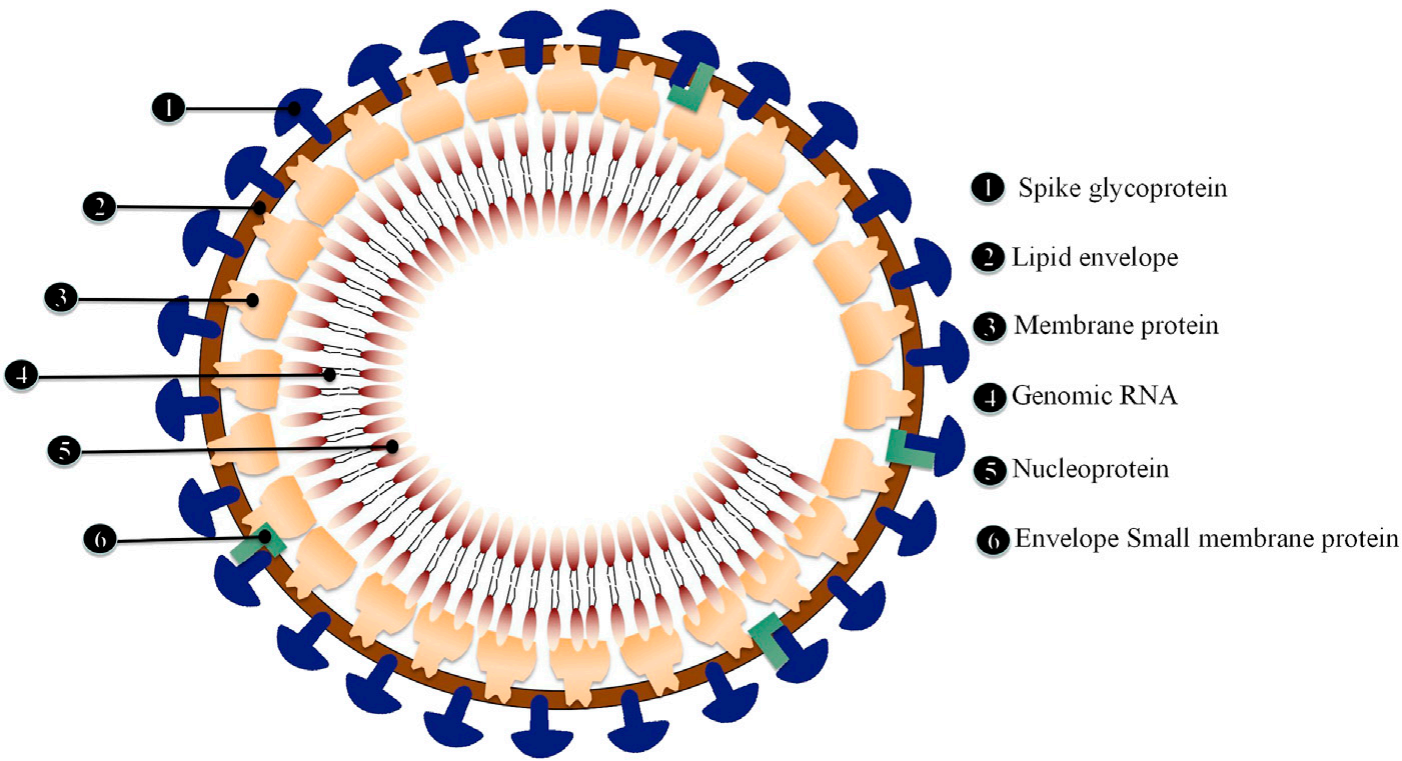
Schematic diagram of SARS-CoV-2 structure.

ACE2 has been identified as the primary receptor for binding SARS-CoV S protein. Researchers have designed and discovered small molecule compounds and peptides that can bind to the SARS-CoV-specific receptor ACE2, preventing SARS-CoV S protein from binding to ACE2 and fusing with the host cell membrane to prevent viral infection (Zhang et al., 2020). This suggests that drugs targeting the virus-acting receptor can be designed. Xu et al. (Xu et al., 2020) confirmed ACE2 as the receptor of SARS-CoV-2 by studying the binding capacity of the structural model of SARS-CoV-2 S protein to human ACE2 receptor. SARS-transmembrane CoV’s spike glycoprotein (S protein) binds to the cellular membrane ACE2; SARS-CoV then attaches to target cells, followed by SARS-CoV-S protein priming by cellular surface proteases such as transmembrane protease serine 2 (TMPRSS2), resulting in the fusion of viral and cellular membranes and SARS-CoV entry and replication in target cells. In addition, elimination of ACE2 reduces viral infection and replication considerably in mice infected with SARS-CoV. It is thus believed that the SARS-CoV S protein’s binding to ACE2 is crucial for SARS-CoV infection (Kai and Kai, 2020). Alveolar epithelial cells bind to SARS-CoV-2. The virus then activates the innate and adaptive immune systems, resulting in a flood of cytokines, including IL-6, being produced (Figure 4).

## Future prospects

In controlled clinical trials throughout the world, IL-6 and IL-6R antagonists are being evaluated for the treatment of COVID-19 patients with severe respiratory difficulties. Unanswered is whether IL-6 antagonists and IL-6R antagonists will have varying degrees of effectiveness. Inhibitors of the IL-6R may disrupt both cis and trans signaling, as well as the recently identified trans presentation signaling. IL-6 binds to mIL-6R on immune cells, which then forms a complex with gp130 on Th17 cells, resulting in T cell signaling that may be involved in ARDS (Tanaka et al., 2016b; Heink et al., 2017; Kang et al., 2019). In contrast, IL-6 inhibitors can only suppress cis and trans signaling. The primary goal of IL-6 antagonistic therapy is to decrease the need for advanced treatment in individuals with severe COVID-19. The development of antivirals and immunizations that prevent or relieve illness should be a long-term goal.

Patients with severe COVID-19, similar to SARS and MERS patients, have been proposed to have a CRS defined by an elevation in IL-6, which indicates that it may aggravate lung damage, cause viral inflammatory response and death. A prior cohort analysis found that IL-6 expression was substantially higher in COVID-19 patients, but that it varied greatly across ICU and non-ICU patients (Liu et al., 2020a). Moreover, recent study has shown that the SARS-CoV S protein promotes an upregulation of IL-6 and TNF in murine macrophages, and IL-6 and IL-8 have been identified as significant SARS-CoV-induced epithelial cytokines (Yoshikawa et al., 2009). These data indicate that SARS-CoV-induced IL-6 and TNF play a role in the disease’s pathogenesis, notably in terms of inflammation and high fever (Wang et al., 2014). Anti-IL6R antibody Tocilizumab is a humanized recombinant monoclonal antibody. Tocilizumab has shown potential for treating severe CRS. Sixty-nine percent of patients responded within 14 days after receiving one or two doses of tocilizumab, with fever and hypotension receding within hours and vasopressors being withdrawn within a few days (Kotch et al., 2019). Tocilizumab’s effect has also been recorded in CRS linked with sepsis, graft-versus-host disease (GvHD) and macrophage activation syndrome (MAS), among others (Barut et al., 2017; Ibrahim et al., 2020; Melgarejo-Ortuno et al., 2021). However, there is not enough evidence to clearly show the clinical efficacy and safety of Tocilizumab for severe patients with COVID-19, and its clinical application and side effects need to be further explored (Cortegiani et al., 2021).

Given the worldwide urgency of containing the COVID-19 pandemic, there are a few cautions to consider. Corticosteroids are often used to treat ARDS caused by sepsis. However, in SARS and MERS patients, corticosteroids did not lower mortality and delayed viral clearance (Channappanavar and Perlman, 2017). Infectious disease authority and the WHO have thus decided that systemic corticosteroids should not be administered to COVID-19 patients at this time. The reduction in inflammation generated by IL-6 antagonism might, in theory, postpone viral clearance. Nevertheless, inhibiting IL-6 induces a rapid drop in serum IL-10, which may assuage concerns over the length of time required for viral clearance (Tanaka et al., 2016b). Furthermore, it is doubtful that one or two doses of an IL-6 antagonist would cause consequences like fungal infections or jaw osteonecrosis, which are common in people using these medications on a monthly basis for chronic illnesses like RA. Tocilizumab was first approved for rheumatic illnesses, then for CRS in patients undergoing CAR-T cell treatment, and is now being repurposed for the COVID-19 pandemic. Diamanti et al. found that compared with HCWs, people using IL-6 inhibitors (such as RA patients) had significantly lower vaccine antibody titers, but almost all patients had antibody specific reactions induced in their bodies. In the investigated RA patients, BNT162b2 vaccine showed good safety (Picchianti-Diamanti et al., 2021).

## Conclusion

As the pandemic grows, experts throughout the globe are working to better understand the virus’s pathophysiology and identify new targets and promising medications that may be used to fight SARS-CoV-2. There are no confirmed antiviral medicines with particular action against SARS-CoV-2, despite some insights about viral pathophysiology and prospective targets. Future pandemics involving more viruses, may use IL-6-targeted treatments Chen et al., 2010, Hu et al., 2021a, Kim et al., 2021, Li et al., 2016, Soy et al., 2020.

## Glossary

ACE2: angiotensin converting enzyme 2
ALI: acute lung injury
AP-1: activator protein 1
ARDS1: acute respiratory distress syndrome 1
CAR-T: chimeric antigen receptor-T
CEST: central european summer time
COVID-19: corona virus disease 2019
CRP: C-reactive protein
CRS: cytokine release syndrome
DAMPs: damage-associated molecular patterns
Gab1: GRB2 associated binding protein 1
GRB2: growth factor receptor-bound protein 2
GvHD: graft-versus-host disease
HCOVs: human coronaviruses
HCWs: health care workers
HMGB1: high mobility group box 1
IL: interleukin
IPC: infection prevention and control
IRF-1: interferon regulatory factor 1
JAK: janus kinase
LPS: lipopolysaccharide
LRT: lower respiratory tract
MAPK: mitogen-activated protein kinase
MAS: macrophage activation syndrome
MERS: middle east respiratory sickness
MERS-CoV: middle east respiratory syndrome coronavirus
mIL-6R: membrane-bound IL6R
MMP-9: matrix metalloproteinase-9
NF-IL-6: nuclear factor IL-6
NF-κB: nuclear factor kappa-B
PAMPs: pathogen-associated molecular patterns
PBMC: peripheral blood mononuclear cell
PCT: procalcitonin
PI3K: phosphatidylinositol 3-kinase
PRRs: pathogen-recognition receptors
RA: rheumatoid arthritis
RAGE: receptor for advanced glycation end products
SAA: serum amyloid A
SARS: severe acute respiratory syndrome
SARS-CoV: severe acute respiratory syndrome coronavirus
SARS-CoV-2: severe acute respiratory syndrome coronavirus 2
SHP-2: Src homology phosphotyrosine phosphatase 2
sIL-6R: soluble IL-6R
SP1: specificity protein 1
TG: thioglycollate
Th17: T helper 17
TLRs: toll-like receptors
TMPRSS2: transmembrane protease serine 2
TNF-α: tumor necrosis factor-α
WHO: World Health Organization

## References

[B1] Akira S. Kishimoto T. (1992). IL-6 and NF-IL6 in acute-phase response and viral infection. Immunol. Rev. 127, 25–50. 10.1111/j.1600-065x.1992.tb01407.x 1380488

[B2] Akira S. Taga T. Kishimoto T. (1993). Interleukin-6 in biology and medicine. Adv. Immunol. 54, 1–78. 10.1016/s0065-2776(08)60532-5 8379461

[B3] Al-Qahtani A. A. Lyroni K. Aznaourova M. Tseliou M. Al-Anazi M. R. Al-Ahdal M. N. (2017). Middle East respiratory syndrome corona virus spike glycoprotein suppresses macrophage responses via DPP4-mediated induction of IRAK-M and PPARγ. Oncotarget 8 (6), 9053–9066. 10.18632/oncotarget.14754 28118607 PMC5354714

[B4] Bai B. Hu Q. Hu H. Zhou P. Shi Z. Meng J. (2008). Virus-like particles of SARS-like coronavirus formed by membrane proteins from different origins demonstrate stimulating activity in human dendritic cells. PLoS One 3 (7), e2685. 10.1371/journal.pone.0002685 18628832 PMC2441860

[B5] Baran P. Hansen S. Waetzig G. H. Akbarzadeh M. Lamertz L. Huber H. J. (2018). The balance of interleukin (IL)-6, IL-6.soluble IL-6 receptor (sIL-6R), and IL-6.sIL-6R.sgp130 complexes allows simultaneous classic and trans-signaling. J. Biol. Chem. 293 (18), 6762–6775. 10.1074/jbc.RA117.001163 29559558 PMC5936821

[B6] Barut K. Adrovic A. Sahin S. Kasapcopur O. (2017). Juvenile idiopathic arthritis. Balk. Med. J. 34 (2), 90–101. 10.4274/balkanmedj.2017.0111 PMC5394305 28418334

[B7] Bianchi M. E. (2007). DAMPs, PAMPs and alarmins: All we need to know about danger. J. Leukoc. Biol. 81 (1), 1–5. 10.1189/jlb.0306164 17032697

[B8] Channappanavar R. Perlman S. (2017). Pathogenic human coronavirus infections: Causes and consequences of cytokine storm and immunopathology. Semin. Immunopathol. 39 (5), 529–539. 10.1007/s00281-017-0629-x 28466096 PMC7079893

[B9] Chen J. Lau Y. F. Lamirande E. W. Paddock C. D. Bartlett J. H. Zaki S. R. (2010). Cellular immune responses to severe acute respiratory syndrome coronavirus (SARS-CoV) infection in senescent BALB/c mice: CD4+ T cells are important in control of SARS-CoV infection. J. Virol. 84 (3), 1289–1301. 10.1128/JVI.01281-09 19906920 PMC2812346

[B10] Chousterman B. G. Swirski F. K. Weber G. F. (2017). Cytokine storm and sepsis disease pathogenesis. Semin. Immunopathol. 39 (5), 517–528. 10.1007/s00281-017-0639-8 28555385

[B11] Cortegiani A. Ippolito M. Greco M. Granone V. Protti A. Gregoretti C. (2021). Rationale and evidence on the use of tocilizumab in COVID-19: A systematic review. Pulmonology 27 (1), 52–66. 10.1016/j.pulmoe.2020.07.003 32713784 PMC7369580

[B12] De Clercq E. (2006). Potential antivirals and antiviral strategies against SARS coronavirus infections. Expert Rev. anti. Infect. Ther. 4 (2), 291–302. 10.1586/14787210.4.2.291 16597209 PMC7105749

[B13] de Wilde A. H. Snijder E. J. Kikkert M. van Hemert M. J. (2018). Host factors in coronavirus replication. Curr. Top. Microbiol. Immunol. 419, 1–42. 10.1007/82_2017_25 28643204 PMC7119980

[B14] de Wit E. van Doremalen N. Falzarano D. Munster V. J. (2016). SARS and MERS: Recent insights into emerging coronaviruses. Nat. Rev. Microbiol. 14 (8), 523–534. 10.1038/nrmicro.2016.81 27344959 PMC7097822

[B15] Declercq J. Van Damme K. F. A. De Leeuw E. Maes B. Bosteels C. Tavernier S. J. (2021). Effect of anti-interleukin drugs in patients with COVID-19 and signs of cytokine release syndrome (COV-aid): A factorial, randomised, controlled trial. Lancet. Respir. Med. 9 (12), 1427–1438. 10.1016/S2213-2600(21)00377-5 34756178 PMC8555973

[B16] Dosch S. F. Mahajan S. D. Collins A. R. (2009). SARS coronavirus spike protein-induced innate immune response occurs via activation of the NF-kappaB pathway in human monocyte macrophages *in vitro* . Virus Res. 142 (1-2), 19–27. 10.1016/j.virusres.2009.01.005 19185596 PMC2699111

[B17] Gillmore J. D. Lovat L. B. Persey M. R. Pepys M. B. Hawkins P. N. (2001). Amyloid load and clinical outcome in AA amyloidosis in relation to circulating concentration of serum amyloid A protein. Lancet 358 (9275), 24–29. 10.1016/S0140-6736(00)05252-1 11454373

[B18] Haffizulla J. Hartman A. Hoppers M. Resnick H. Samudrala S. Ginocchio C. (2014). Effect of nitazoxanide in adults and adolescents with acute uncomplicated influenza: A double-blind, randomised, placebo-controlled, phase 2b/3 trial. Lancet. Infect. Dis. 14 (7), 609–618. 10.1016/S1473-3099(14)70717-0 24852376 PMC7164783

[B19] Hamre D. Procknow J. J. (1966). A new virus isolated from the human respiratory tract. Proc. Soc. Exp. Biol. Med. 121 (1), 190–193. 10.3181/00379727-121-30734 4285768

[B20] He L. Ding Y. Zhang Q. Che X. He Y. Shen H. (2006). Expression of elevated levels of pro-inflammatory cytokines in SARS-CoV-infected ACE2+ cells in SARS patients: Relation to the acute lung injury and pathogenesis of SARS. J. Pathol. 210 (3), 288–297. 10.1002/path.2067 17031779 PMC7167655

[B21] Heink S. Yogev N. Garbers C. Herwerth M. Aly L. Gasperi C. (2017). Corrigendum: Trans-presentation of IL-6 by dendritic cells is required for the priming of pathogenic TH17 cells. Nat. Immunol. 18 (1), 474–485. 10.1038/ni0417-474b 28323263

[B22] Heinrich P. C. Behrmann I. Haan S. Hermanns H. M. Muller-Newen G. Schaper F. (2003). Principles of interleukin (IL)-6-type cytokine signalling and its regulation. Biochem. J. 374 (1), 1–20. 10.1042/BJ20030407 12773095 PMC1223585

[B23] Heinrich P. C. Castell J. V. Andus T. (1990). Interleukin-6 and the acute phase response. Biochem. J. 265 (3), 621–636. 10.1042/bj2650621 1689567 PMC1133681

[B24] Hijawi B. Abdallat M. Sayaydeh A. Alqasrawi S. HaddAdin A. JaarourN. (2013). Novel coronavirus infections in Jordan, April 2012: Epidemiological findings from a retrospective investigation. East. Mediterr. Health J. 19 (1), S12–S18. 10.26719/2013.19.supp1.s12 23888790

[B25] Hsueh P. R. Chen P. J. Hsiao C. H. Yeh S. H. Cheng W. C. Wang J. L. (2004). Patient data, early SARS epidemic, Taiwan. Emerg. Infect. Dis. 10 (3), 489–493. 10.3201/eid1003.030571 15109419

[B26] Hu B. Huang S. Yin L. (2021). The cytokine storm and COVID-19. J. Med. Virol. 93 (1), 250–256. 10.1002/jmv.26232 32592501 PMC7361342

[B27] Hu S. Li Z. Chen X. Liang C. H. (2021). Computed tomography manifestations in super early stage 2019 novel coronavirus pneumonia. Acta Radiol. 62 (3), 360–367. 10.1177/0284185120924806 32438876 PMC7930599

[B28] Huang C. Wang Y. Li X. Ren L. Zhao J. Hu Y. (2020). Clinical features of patients infected with 2019 novel coronavirus in Wuhan, China. Lancet 395 (10223), 497–506. 10.1016/S0140-6736(20)30183-5 31986264 PMC7159299

[B29] Ibrahim Y. F. Moussa R. A. Bayoumi A. M. A. Ahmed A. F. (2020). Tocilizumab attenuates acute lung and kidney injuries and improves survival in a rat model of sepsis via down-regulation of NF-κB/JNK: A possible role of P-glycoprotein. Inflammopharmacology 28 (1), 215–230. 10.1007/s10787-019-00628-y 31440860

[B30] Jiang Z. Liao R. Lv J. Li S. Zheng D. Qin L. (2021). IL-6 trans-signaling promotes the expansion and anti-tumor activity of CAR T cells. Leukemia 35 (5), 1380–1391. 10.1038/s41375-020-01085-1 33168950

[B31] Jones B. M. Ma E. S. Peiris J. S. Wong P. C. Ho J. C. M. Lam B. (2004). Prolonged disturbances of *in vitro* cytokine production in patients with severe acute respiratory syndrome (SARS) treated with ribavirin and steroids. Clin. Exp. Immunol. 135 (3), 467–473. 10.1111/j.1365-2249.2003.02391.x 15008980 PMC1808981

[B32] Kai H. Kai M. (2020). Interactions of coronaviruses with ACE2, angiotensin II, and RAS inhibitors-lessons from available evidence and insights into COVID-19. Hypertens. Res. 43 (7), 648–654. 10.1038/s41440-020-0455-8 32341442 PMC7184165

[B33] Kang S. Tanaka T. Kishimoto T. (2015). Therapeutic uses of anti-interleukin-6 receptor antibody. Int. Immunol. 27 (1), 21–29. 10.1093/intimm/dxu081 25142313

[B34] Kang S. Tanaka T. Narazaki M. Kishimoto T. (2019). Targeting interleukin-6 signaling in clinic. Immunity 50 (4), 1007–1023. 10.1016/j.immuni.2019.03.026 30995492

[B35] Kaur S. Bansal Y. Kumar R. Bansal G. (2020). A panoramic review of IL-6: Structure, pathophysiological roles and inhibitors. Bioorg. Med. Chem. 28 (5), 115327. 10.1016/j.bmc.2020.115327 31992476

[B36] Kim J. S. Lee J. Y. Yang J. W. Lee K. H. Effenberger M. Szpirt W. (2021). Immunopathogenesis and treatment of cytokine storm in COVID-19. Theranostics 11 (1), 316–329. 10.7150/thno.49713 33391477 PMC7681075

[B37] Kishimoto T. (2010). IL-6: From its discovery to clinical applications. Int. Immunol. 22 (5), 347–352. 10.1093/intimm/dxq030 20410258

[B38] Korea Centers for Disease C. (2015). Prevention Middle East respiratory syndrome coronavirus outbreak in the republic of Korea, 2015. Osong Public Health Res. Perspect. 6 (4), 269–278. 26473095 10.1016/j.phrp.2015.08.006 PMC4588443

[B39] Kotch C. Barrett D. Teachey D. T. (2019). Tocilizumab for the treatment of chimeric antigen receptor T cell-induced cytokine release syndrome. Expert Rev. Clin. Immunol. 15 (8), 813–822. 10.1080/1744666X.2019.1629904 31219357 PMC7936577

[B40] Kujawski M. Kortylewski M. Lee H. Herrmann A. Kay H. Yu H. (2008). Stat3 mediates myeloid cell-dependent tumor angiogenesis in mice. J. Clin. Investig. 118 (10), 3367–3377. 10.1172/JCI35213 18776941 PMC2528912

[B41] Kumar H. Kawai T. Akira S. (2011). Pathogen recognition by the innate immune system. Int. Rev. Immunol. 30 (1), 16–34. 10.3109/08830185.2010.529976 21235323

[B42] Lagunas-Rangel F. A. Chavez-Valencia V. (2020). High IL-6/IFN-gamma ratio could be associated with severe disease in COVID-19 patients. J. Med. Virol. 92 (10), 1789–1790. 10.1002/jmv.25900 32297995 PMC7262117

[B43] Lai X. Wang M. Qin C. Tan L. Ran L. Chen D. (2020). Coronavirus disease 2019 (COVID-2019) infection among health care workers and implications for prevention measures in a tertiary hospital in wuhan, China. JAMA Netw. Open 3 (5), e209666. 10.1001/jamanetworkopen.2020.9666 32437575 PMC7243089

[B44] Lee D. W. Gardner R. Porter D. L. Louis C. U. Ahmed N. Jensen M. (2014). Current concepts in the diagnosis and management of cytokine release syndrome. Blood 124 (2), 188–195. 10.1182/blood-2014-05-552729 24876563 PMC4093680

[B45] Li S. W. Wang C. Y. Jou Y. J. Huang S. H. Hsiao L. H. Wan L. (2016). SARS coronavirus papain-like protease inhibits the TLR7 signaling pathway through removing lys63-linked polyubiquitination of TRAF3 and TRAF6. Int. J. Mol. Sci. 17 (5), E678. 10.3390/ijms17050678 PMC4881504 27164085

[B46] Libermann T. A. Baltimore D. (1990). Activation of interleukin-6 gene expression through the NF-kappa B transcription factor. Mol. Cell. Biol. 10 (5), 2327–2334. 10.1128/mcb.10.5.2327 2183031 PMC360580

[B47] Liu B. Li M. Zhou Z. Guan X. Xiang Y. (2020). Can we use interleukin-6 (IL-6) blockade for coronavirus disease 2019 (COVID-19)-induced cytokine release syndrome (CRS)? J. Autoimmun. 111, 102452. 10.1016/j.jaut.2020.102452 32291137 PMC7151347

[B48] Liu F. Li L. Xu M. Wu J. Luo D. Zhu Y. (2020). Prognostic value of interleukin-6, C-reactive protein, and procalcitonin in patients with COVID-19. J. Clin. Virol. 127, 104370. 10.1016/j.jcv.2020.104370 32344321 PMC7194648

[B49] Luo X. Zhou W. Yan X. Guo T. Wang B. Xia H. (2020). Prognostic value of C-reactive protein in patients with coronavirus 2019. Clin. Infect. Dis. 71 (16), 2174–2179. 10.1093/cid/ciaa641 32445579 PMC7314209

[B50] Mackay I. M. Arden K. E. (2015). MERS coronavirus: Diagnostics, epidemiology and transmission. Virol. J. 12, 222. 10.1186/s12985-015-0439-5 26695637 PMC4687373

[B51] Matsusaka T. Fujikawa K. Nishio Y. MukaidaN. Matsushima K. KishimoTo T. (1993). Transcription factors NF-IL6 and NF-kappa B synergistically activate transcription of the inflammatory cytokines, interleukin 6 and interleukin 8. Proc. Natl. Acad. Sci. U. S. A. 90 (21), 10193–10197. 10.1073/pnas.90.21.10193 8234276 PMC47740

[B52] McIntosh K. Dees J. H. Becker W. B. Kapikian A. Z. Chanock R. M. (1967). Recovery in tracheal organ cultures of novel viruses from patients with respiratory disease. Proc. Natl. Acad. Sci. U. S. A. 57 (4), 933–940. 10.1073/pnas.57.4.933 5231356 PMC224637

[B53] Melgarejo-Ortuno A. Escudero-Vilaplana V. Revuelta-Herrero J. L. Bailen R. Collado-Borrell R. Gomez-Centurion I. (2021). Tocilizumab as salvage treatment of refractory pulmonary acute graft-versus-host disease. J. Oncol. Pharm. Pract. 27 (3), 751–755. 10.1177/1078155220948934 32787560

[B54] Mihara M. Hashizume M. Yoshida H. Suzuki M. Shiina M. (2012). IL-6/IL-6 receptor system and its role in physiological and pathological conditions. Clin. Sci. 122 (4), 143–159. 10.1042/CS20110340 22029668

[B55] Moore J. B. June C. H. (2020). Cytokine release syndrome in severe COVID-19. Science 368 (6490), 473–474. 10.1126/science.abb8925 32303591

[B56] Nishimoto N. Yoshizaki K. Tagoh H. MondenM. KiShimoto S. Hirano T. (1989). Elevation of serum interleukin 6 prior to acute phase proteins on the inflammation by surgical operation. Clin. Immunol. Immunopathol. 50 (3), 399–401. 10.1016/0090-1229(89)90147-5 2465111

[B57] Norelli M. Camisa B. Barbiera G. Falcone L. Purevdorj A. Genua M. (2018). Monocyte-derived IL-1 and IL-6 are differentially required for cytokine-release syndrome and neurotoxicity due to CAR T cells. Nat. Med. 24 (6), 739–748. 10.1038/s41591-018-0036-4 29808007

[B58] Pandolfi F. Franza L. Carusi V. Altamura S. Andriollo G. Nucera E. (2020). Interleukin-6 in rheumatoid arthritis. Int. J. Mol. Sci. 21 (15), E5238. 10.3390/ijms21155238 PMC7432115 32718086

[B59] Picchianti-Diamanti A. Aiello A. Lagana B. Agrati C. Castilletti C. Meschi S. (2021). ImmunosuppressiveTherapies differently modulate humoral- and T-cell-specific responses to COVID-19 mRNA vaccine in rheumatoid arthritis patients. Front. Immunol. 12, 740249. Supplementary Materials. 10.3389/fimmu.2021.740249 34594343 PMC8477040

[B60] Rossignol J. F. La Frazia S. Chiappa L. Ciucci A. Santoro M. G. (2009). Thiazolides, a new class of anti-influenza molecules targeting viral hemagglutinin at the post-translational level. J. Biol. Chem. 284 (43), 29798–29808. 10.1074/jbc.M109.029470 19638339 PMC2785610

[B61] Rossignol J. F. (2016). Nitazoxanide, a new drug candidate for the treatment of Middle East respiratory syndrome coronavirus. J. Infect. Public Health 9 (3), 227–230. 10.1016/j.jiph.2016.04.001 27095301 PMC7102735

[B62] Sheng W. H. Chiang B. L. Chang S. C. Ho H. N. Wang J. T. Chen Y. C. (2005). Clinical manifestations and inflammatory cytokine responses in patients with severe acute respiratory syndrome. J. Formos. Med. Assoc. 104 (10), 715–723. 16385373

[B63] Shimabukuro-Vornhagen A. Godel P. Subklewe M. Stemmler H. J. SchloBer H. A. Schlaak M. (2018). Cytokine release syndrome. J. Immunother. Cancer 6 (1), 56. 10.1186/s40425-018-0343-9 29907163 PMC6003181

[B64] Sims G. P. Rowe D. C. Rietdijk S. T. Herbst R. Coyle A. J. (2010). HMGB1 and RAGE in inflammation and cancer. Annu. Rev. Immunol. 28, 367–388. 10.1146/annurev.immunol.021908.132603 20192808

[B65] Smilowitz N. R. Kunichoff D. Garshick M. Shah B. Pillinger M. Hochman J. S. (2021). C-reactive protein and clinical outcomes in patients with COVID-19. Eur. Heart J. 42 (23), 2270–2279. 10.1093/eurheartj/ehaa1103 33448289 PMC7928982

[B66] Soin A. S. Kumar K. Choudhary N. S. Sharma P. Mehta Y. Kataria S. (2021). Tocilizumab plus standard care versus standard care in patients in India with moderate to severe COVID-19-associated cytokine release syndrome (COVINTOC): An open-label, multicentre, randomised, controlled, phase 3 trial. Lancet. Respir. Med. 9 (5), 511–521. 10.1016/S2213-2600(21)00081-3 33676589 PMC8078880

[B67] Soy M. Keser G. Atagunduz P. Tabak F. Atagunduz I. Kayhan S. (2020). Cytokine storm in COVID-19: Pathogenesis and overview of anti-inflammatory agents used in treatment. Clin. Rheumatol. 39 (7), 2085–2094. 10.1007/s10067-020-05190-5 32474885 PMC7260446

[B68] Stone J. H. Frigault M. J. Serling-Boyd N. J. Fernandes A. D. Harvey L. Foulkes A. S. (2020). Efficacy of tocilizumab in patients hospitalized with covid-19. N. Engl. J. Med. 383 (24), 2333–2344. 10.1056/NEJMoa2028836 33085857 PMC7646626

[B69] Tanaka T. Kishimoto T. (2014). The biology and medical implications of interleukin-6. Cancer Immunol. Res. 2 (4), 288–294. 10.1158/2326-6066.CIR-14-0022 24764575

[B70] Tanaka T. Narazaki M. Kishimoto T. (2014). IL-6 in inflammation, immunity, and disease. Cold Spring Harb. Perspect. Biol. 6 (10), a016295. 10.1101/cshperspect.a016295 25190079 PMC4176007

[B71] Tanaka T. Narazaki M. Kishimoto T. (2016). Immunotherapeutic implications of IL-6 blockade for cytokine storm. Immunotherapy 8 (8), 959–970. 10.2217/imt-2016-0020 27381687

[B72] Tanaka T. Narazaki M. Kishimoto T. (2012). Therapeutic targeting of the interleukin-6 receptor. Annu. Rev. Pharmacol. Toxicol. 52, 199–219. 10.1146/annurev-pharmtox-010611-134715 21910626

[B73] Tanaka T. Narazaki M. Masuda K. Kishimoto T. (2016). Regulation of IL-6 in immunity and diseases. Adv. Exp. Med. Biol. 941, 79–88. 10.1007/978-94-024-0921-5_4 27734409

[B74] Teijaro J. R. (2017). Cytokine storms in infectious diseases. Semin. Immunopathol. 39 (5), 501–503. 10.1007/s00281-017-0640-2 28674818 PMC7079934

[B75] van der Hoek L. (2007). Human coronaviruses: What do they cause? Antivir. Ther. 12 (4), 651–658. 10.1177/135965350701200s01.1 17944272

[B76] van der Hoek L. Pyrc K. Jebbink M. F. Vermeulen-Oost W. Berkhout R. J. M. Wolthers K. C. (2004). Identification of a new human coronavirus. Nat. Med. 10 (4), 368–373. 10.1038/nm1024 15034574 PMC7095789

[B77] Wang C. Fei D. Li X. Zhao M. Yu K. (2020). IL-6 may be a good biomarker for earlier detection of COVID-19 progression. Intensive Care Med. 46 (7), 1475–1476. 10.1007/s00134-020-06065-8 32385523 PMC7206216

[B78] Wang S. F. Tseng S. P. Yen C. H. Yang J. Y. Tsao C. H. Shen C. W. (2014). Antibody-dependent SARS coronavirus infection is mediated by antibodies against spike proteins. Biochem. Biophys. Res. Commun. 451 (2), 208–214. 10.1016/j.bbrc.2014.07.090 25073113 PMC7092860

[B79] Wise J. (2012). Patient with new strain of coronavirus is treated in intensive care at London hospital. BMJ 345, e6455. 10.1136/bmj.e6455 23008211

[B80] Woo P. C. Lau S. K. Chu C. M. Chan K. h. Tsoi H. w. Huang Y. (2005). Characterization and complete genome sequence of a novel coronavirus, coronavirus HKU1, from patients with pneumonia. J. Virol. 79 (2), 884–895. 10.1128/JVI.79.2.884-895.2005 15613317 PMC538593

[B81] Xu X. Chen P. Wang J. Feng J. Zhou H. Li X. (2020). Evolution of the novel coronavirus from the ongoing Wuhan outbreak and modeling of its spike protein for risk of human transmission. Sci. China. Life Sci. 63 (3), 457–460. 10.1007/s11427-020-1637-5 32009228 PMC7089049

[B82] Yoshikawa T. Hill T. Li K. Peters C. J. Tseng C. T. (2009). Severe acute respiratory syndrome (SARS) coronavirus-induced lung epithelial cytokines exacerbate SARS pathogenesis by modulating intrinsic functions of monocyte-derived macrophages and dendritic cells. J. Virol. 83 (7), 3039–3048. 10.1128/JVI.01792-08 19004938 PMC2655569

[B83] Yu H. Pardoll D. Jove R. (2009). STATs in cancer inflammation and immunity: A leading role for STAT3. Nat. Rev. Cancer 9 (11), 798–809. 10.1038/nrc2734 19851315 PMC4856025

[B84] Yu P. Hu B. Shi Z. L. Cui J. (2019). Geographical structure of bat SARS-related coronaviruses. Infect. Genet. Evol. 69, 224–229. 10.1016/j.meegid.2019.02.001 30735813 PMC7106260

[B85] Yu S. Y. Hu Y. W. Liu X. Y. Xiong W. Zhou Z. T. Yuan Z. H. (2005). Gene expression profiles in peripheral blood mononuclear cells of SARS patients. World J. Gastroenterol. 11 (32), 5037–5043. 10.3748/wjg.v11.i32.5037 16124062 PMC4321926

[B86] Zaki A. M. van Boheemen S. Bestebroer T. M. Osterhaus A. D. Fouchier R. A. (2012). Isolation of a novel coronavirus from a man with pneumonia in Saudi Arabia. N. Engl. J. Med. 367 (19), 1814–1820. 10.1056/NEJMoa1211721 23075143

[B87] Zhang X. Li S. Niu S. (2020). ACE2 and COVID-19 and the resulting ARDS. Postgrad. Med. J. 96 (1137), 403–407. 10.1136/postgradmedj-2020-137935 32522846 PMC10016912

[B88] Zhang Z. W. Zhang Q. Y. Zhou M. T. Liu N. X. Chen T. K. Zhu Y. F. (2010). Antioxidant inhibits HMGB1 expression and reduces pancreas injury in rats with severe acute pancreatitis. Dig. Dis. Sci. 55 (9), 2529–2536. 10.1007/s10620-009-1073-0 19997973

