# Supplementary material for: The role of IL-6 in coronavirus, especially in COVID-19
Source: Front Pharmacol. 2022 Nov 23;13:1033674. doi: 10.3389/fphar.2022.1033674 (PMC9727200; doi:10.3389/fphar.2022.1033674)
Supplement: Supplementary file 1 [file Table1.docx]

| **Cytokines** | **SARS-COV-2** | | **SARS-COV** | | **MERS-COV** | |
| --- | --- | --- | --- | --- | --- | --- |
| **IL-6** | **↑↑** | **32301997** | **↑↑** | **17532082** | **↑** | **24065148** |
| **TNF-α** | **↑** | **32217835** | **↑** | **17532082 18312678** | **↑** | **29414327** |
| **IL-1β** | **↑** | **32222466** | **NA** | **21324206** | **↑↑** | **30634407** |
| **IL-4** | **↑** | **32007143** | **↑** | **15008980** | **NA** | **29414327** |
| **IL-2** | **↑** | **32217835** | **↑** | **15008980** | **NA** | **29414327** |
| **IL-8** | **↑** | **32217835** | **↑** | **22876772** | **↑** | **24065148** |
| **IL-10** | **↑** | **32475230** | **↑** | **15008980** | **↑** | **28118607** |
| **IL-15** | **Unknown** |  | **↓(Monkey)** | **24642138** | **↑** | **29414327** |
| **IL-17** | **↑** | **32534467** | **↑** | **21964025** | **↑** | **29414327 32352535** |
| **IP10** | **↑** | **32161940** | **↑** | **17243893** | **↑↑** | **25547683** |
| **MCP-1** | **↑** | **32534467** | **↑** | **19853271** | **↑** | **24065148** |
| **IFN-γ** | **↑** | **31986264** | **↑** | **15030519** | **↑↑** | **29414327 25547683** |
